# Factors influencing community-facility linkage for case management of possible serious bacterial infections among young infants in Kenya

**DOI:** 10.1093/heapol/czad113

**Published:** 2023-11-29

**Authors:** George Odwe, Wilson Liambila, Kezia K’Oduol, Zipporah Nyangacha, Helen Gwaro, Alexandra Haake Kamberos, Lisa R Hirschhorn

**Affiliations:** Population Council Kenya, P.O Box 17643, Nairobi 00500, Kenya; Population Council Kenya, P.O Box 17643, Nairobi 00500, Kenya; Living Goods-Kenya, P.O. Box 30261, Nairobi 00100, Kenya; Living Goods-Kenya, P.O. Box 30261, Nairobi 00100, Kenya; Lwala Community Alliance, P.O. Box 24, Rongo 40404, Kenya; Northwestern University, Feinberg School of Medicine and Havey Institute of Global Health, 625 North Michigan Ave, 14-013, Chicago, IL 60611, United States; Northwestern University, Feinberg School of Medicine and Havey Institute of Global Health, 625 North Michigan Ave, 14-013, Chicago, IL 60611, United States

**Keywords:** PSBI continuum of care, community, facility, linkage

## Abstract

Despite evidence showing the feasibility and acceptability of implementing the World Health Organization’s guidelines on managing possible serious bacterial infection (PSBI) in Kenya, the initial implementation revealed sub-optimal community-facility referrals and follow-up of PSBI cases. This study explores facilitators and barriers of community-facility linkages in implementing PSBI guidelines in Busia and Migori counties, Kenya. We used an exploratory qualitative study design drawing on endline evaluation data from the ‘COVID-19: Mitigating Neonatal Mortality’ project collected between June and July 2022. Data include case narratives with caregivers of sick young infants (0–59 days old) (18), focus group discussions with community health volunteers (CHVs) (6), and in-depth interviews with facility-based providers (18). Data were analysed using an inductive thematic analysis framework. Between August 2021 and July 2022, CHVs assessed 10 187 newborns, with 1176 (12%) identified with PSBI danger signs and referred to the nearest facility, of which 820 (70%) accepted referral. Analysis revealed several factors facilitating community-facility linkage for PSBI treatment, including CHVs’ relationship with community members and facilities, availability of a CHV desk and tools, use of mobile app, training and supportive supervision. However, challenges such as health system-related factors (inadequate providers, stockout of essential commodities and supplies, and lack of transport/ambulance) and individual-related factors (caregivers’ refusal to take referrals) hindered community-facility linkage. Addressing common barriers and fostering positive relationships between community health workers and facilities can enhance acceptance and access to PSBI services at the community level. Combining community health workers’ efforts with a mobile digital strategy can improve the efficiency of the identification, referral and tracking of PSBI cases in the community and facilitate linkage with primary healthcare facilities.

Key messages

- Strengthening capacity of community health workers through training, supervision and incentives facilitates prompt identification of sick young infants with possible serious bacterial infection (PSBI) in the community and linkage to health facilities for care.
- Combining community health workers’ efforts with a mobile digital strategy can improve the continuum of PSBI care—identification, referral and follow up.
- Community health link desk at primary health facilities improves sick young infants tracking and follow-up.

## Introduction

Neonatal sepsis, also known as possible serious bacterial infection (PSBI), is a major cause of infant mortality in Sub-Saharan Africa (SSA), accounting for 37% of neonatal deaths each year (Ahmed *et al.*, 2018). Despite notable progress in reducing under-five mortality in Kenya over the past two decades, progress in the neonatal mortality rate (NMR) has been modest and remains high at 21 deaths per 1000 live births against the national and global target of 12 deaths per 1000 live births (KNBS & ICF, 2023). To address this gap, Kenya adopted the World Health Organization (WHO) guidelines for outpatient management of PSBI among infants (0–59 days) in primary healthcare facilities (PHCFs) when a referral is not feasible (WHO, 2015). The PSBI guidelines are implemented as part of the integrated management of newborn and childhood illness (IMNCI) (MOH, 2018).

At the community level, referral of sick newborns has been emphasized in the Integrated Community Case Management (iCCM) strategy, an approach aimed at reducing morbidity and mortality in the under-five population by delivering high-quality services to hard-to-reach populations (Shiroya-Wandabwa *et al.*, 2018). The success of the PSBI/iCCM strategy hinges on establishing a strong linkage between communities and PHCFs. In Kenya, Community Health Workers (CHWs), also known as Community Health Volunteers (CHVs), whether paid or volunteer, play an important role in bridging community and clinical services (MOH, 2013B). The community-clinical linkage model is layered upon a functional community health system that leverages and links with the network of PHCFs (Lohr *et al.*, 2018; Avan *et al.*, 2021). The role of CHVs in the PSBI ‘care cascade’ or ‘continuum of care’, involves multiple necessary steps, including identification of danger signs in newborns during home visits, referral to the nearest PHCFs and conducting follow-ups in homes to ensure treatment and adherence to PSBI management protocol (Khanal *et al.*, 2011).

Despite evidence showing the feasibility and acceptability of implementing the WHO’s PSBI guidelines in Kenya, initial implementation found sub-optimal community-facility referrals and follow-up of PSBI cases (Odwe *et al.*, 2020; Mbugua *et al.*, 2021; Liambila *et al.*, 2023). The sub-optimal performance is attributed to factors such as weak linkages between communities and health facilities, inadequate skills among community- and facility-based health workers and limited knowledge of the causes of young infant illnesses among community members, which hinders optimal PSBI treatment coverage (Odwe *et al.*, 2020; Liambila *et al.*, 2023). Studies have also highlighted the importance of timely home visits, CHWs’ relationship with community members and supportive supervision to ensure the continuum and quality of PSBI care (Give *et al.*, 2019; Kerebih *et al.*, 2021). Other barriers to community-facility linkage for the management of PSBI include health-system factors such as inadequately trained staff, lack of ambulance services and stockout of commodities (Geletu *et al.*, 2015) and context-specific factors such as cultural beliefs and practices (Nigatu *et al.*, 2015; Odwe *et al.*, 2020). The COVID-19 pandemic exacerbated some of these challenges, causing further setbacks and laying bare the precariousness of health systems in many low- and middle-income countries, including Kenya (Kimani *et al.*, 2020; Oluoch-Aridi *et al.*, 2020).

Evidence highlights the lessons learnt from WHO’s PSBI guidelines implementation in low-resource settings (Applegate *et al.*, 2019; Mukhopadhyay *et al.*, 2021; Nisar *et al.*, 2022). However, there exists limited understanding of the facilitators and barriers to community-facility linkage for the management of PSBI in sick young infants (SYIs) when a referral is not feasible in Kenya. This study explored facilitators and barriers to community-facility linkages for the case management of PSBI among SYIs (0–59 days old) in Busia and Migori counties in Kenya. Understanding facilitators and barriers to community-facility linkages for the case management of PSBI is important for informing newborn and child health interventions.

## Methods

### Study design

Data were from an endline evaluation of the ‘*COVID-19: Mitigating Neonatal Mortality’* project (also known as the PSBI II project)—an implementation science project aimed at testing and adapting strategies to improve the uptake of PSBI guidelines during the COVID-19 pandemic in Kenya. The project was implemented between December 2020 and August 2022 and employed a hybrid II implementation research design drawing on the Research Effectiveness Adoption Implementation Maintenance (RE-AIM) framework (Glasgow *et al.*, 1999). The PSBI II project followed a before-and-after approach with multiple data sources over three distinct phases: formative, routine information feedback, monitoring and an endline evaluation. This paper is based on PSBI II programme monitoring data and endline qualitative data: focus group discussion (FGDs) with CHVs, case narratives with caregivers SYIs (0–59 days old) and in-depth interviews (IDIs) with facility-based health providers.

### Context and study setting

The study was implemented in two counties: Busia (in Teso North and Bunyala sub-counties) and Migori (in Rongo sub-county) in western Kenya. These sites were selected in consultation with the county health management teams. Both counties have high maternal mortality ratio (MMR), NMRs and fertility rates. Migori County has a MMR of 673 deaths per 100 000 live births, a total fertility rate of 5.3 and NMR that mirrors the national patterns (Afidep, 2017). The under-five mortality in Rongo is estimated at 53/1000 live births, (Starnes *et al.*, 2018). On the other hand, Busia County has a MRR of 307 deaths/1000 births (Wafula *et al.*, 2020), total fertility rate of 4.7 and NMR of 24 deaths per 1000 live births (KNBS, MOH, NACC, KEMRI & NCPD, 2015).

The project sites included 39 public and private PHCFs (10 in Rongo, 10 in Bunyala and 19 in Teso North sub-counties) and their surrounding community health units (CHU), which is the first level of the health system. CHU consists of Community Health Committees, Community Health Assistants or Community Health Officers and CHVs. CHVs are members of the community nominated from within, who are tasked with improving the community’s health and well-being and linking individuals to primary health care services (MOH, 2013a). The PSBI II project implementing partners supported CHVs within the existing government community health structure in Busia and Migori counties. This support included training, support supervision, provision of tools, including digital resources and incentives.

### Intervention

The intervention involved training CHVs to provide health education and counsel mothers about newborn care, including identification of sick infants (0–59 days) with PSBI symptoms using a checklist approved by the Ministry of Health (MOH). Trained CHVs conducted home visits to counsel women and their families during pregnancy and postnatal period. During such visits, CHVs used caregiver’s pamphlets to counsel families about essential newborn care practices, recognition of danger signs and prompt care-seeking. Details of each family member were registered on the mobile app to ease tracking. CHVs conducted postnatal care visits within 48 hours, on days 3 and 7 after birth to promote essential newborn care and to assess mothers and young infants for signs of illness. Newborns were followed for up to two months of age to ensure they received relevant PSBI/iCCM services. If a danger sign was identified, the SYI was referred to the nearby PHCF for further assessment and management. If a referral was accepted, CHVs filled MOH 100 form (a community referral form indicating symptoms diagnosed and any interventions done to the SYI by a CHV), in triplicate. In case of a refusal, CHVs re-emphasized the need for referral.

**Figure 1.**
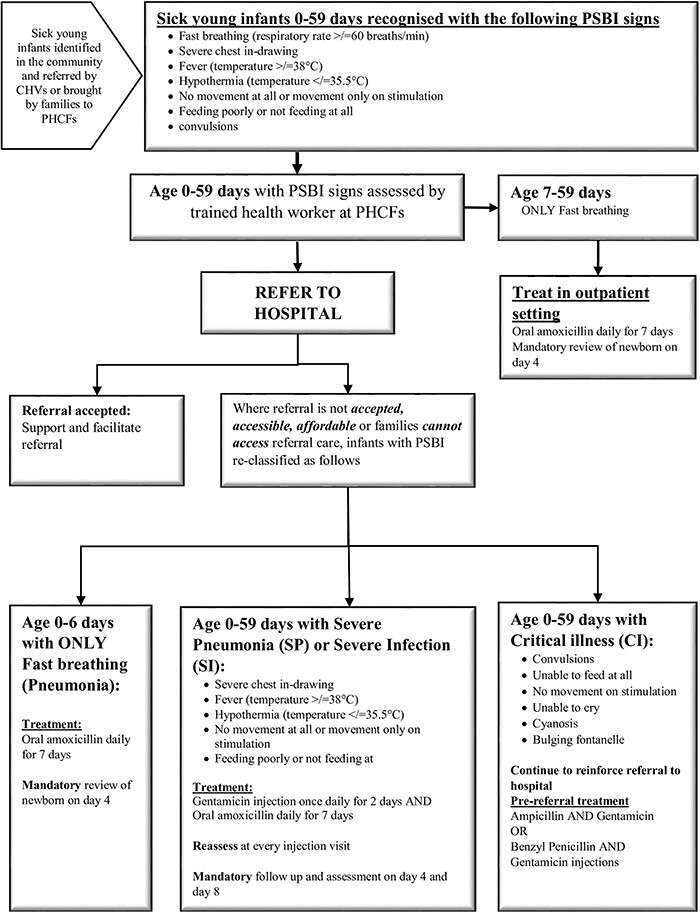
Integrated management of newborn and childhood illnesses (IMNCI)/possible serious bacterial infection (PSBI) flow chart (MOH, 2018)

At the PHCF, providers re-assessed SYIs referred by CHVs or brought by their families for signs of PSBI based on the IMNCI/PSBI guidelines. Those requiring in-patient care at a higher-level facility were referred. Whenever a referral was not feasible, SYIs with PSBI signs were re-classified into either fast-breathing (pneumonia), severe pneumonia/severe infection or critical illness and managed as per the MOH’s IMNCI/PSBI algorithm (Figure 1). CHVs, using a mobile app, tracked referred SYIs, monitored their treatment schedules and return date(s) to the facility for re-assessment. Moreover, CHVs conducted home visits to encourage completion of injections and oral antibiotics as well as to return to the health facility for follow-up on days 2, 4, and 8, where applicable.

### Target population

The study primary target population were caregivers to SYIs, frontline health providers working in PHCFs and CHVs.

### Data collection

The endline survey was conducted between June and July 2022. Data were collected through case narratives with caregivers of SYIs (0–59 days old) (18), FGDs with CHVs (6) and IDIs with facility-based providers (18) as shown in Table 1.

**Table 1.** Data collection methods

Data collection technique		Rongo sub-county (Migori)	Bunyala sub-county (Busia)	Teso-North sub-county (Busia)	Total
Case narratives (CN)		6	6	6	18
Focus group discussions (FGDs)		2	2	2	6
In-depth interviews (IDIs)	Hospitals	2	2	2	6
	Health centres	2	2	2	6
	Dispensaries	2	2	2	6

#### Case narrative

Eighteen case narratives (6 per sub-county) were conducted with caregivers who sought PSBI care over the two months preceding endline data collection. Case narratives explored caregivers’ experiences in seeking care for PSBI cases. Caregivers were selected through convenience sampling, targeting mothers aged 18–49 years of infants (0–59 days old), willing and able to share care-seeking experiences for infant illness. Eligible participants were identified from participating health facilities with the help of providers after they sought care, and in the community with the help of CHVs. Case narratives were conducted in Kiswahili or the local language, using a guide with pre-identified themes, and were audio-recorded by research assistants experienced in qualitative research after obtaining written consent from participants.

#### FGDs with CHVs

At the endline, six FGDs were conducted with CHVs (2 FGDs per sub-county). The FGDs were of mixed gender and comprised eight to ten participants, purposively identified based on their roles in the interventions. Using the FGD guide, research assistants moderated the discussions in Kiswahili after obtaining written consent from participants. The discussions captured CHVs’ opinions regarding the provision of SYI healthcare services in the community, referral services, adherence to PSBI treatment protocol by caregivers, training and supervision needs, and views on how to strengthen community and facility links for PSBI management. All discussions were audio-recorded with the consent of participants.

#### IDIs with facility-based healthcare providers

At the endline, a total of 18 IDIs were conducted with facility-based providers. The IDIs explored providers’ knowledge and experience providing SYI services. Specifically, interviews explored provider awareness, use of IMNCI/PSBI guideline and experience providing IMNCI/PSBI services to SYIs (including PSBI identification, classification, treatment, and referral services). In addition, the IDIs explored providers’ views regarding the quality of SYI healthcare services and how to strengthen community and facility links for PSBI management. Providers were purposely selected to represent different levels of care (hospitals, health centres, and dispensaries/clinics). The interviews were conducted in English and were audio-recorded by research assistants after obtaining written consent from participants, using a guide with pre-identified themes.

### Data analysis

Audio-recorded interviews were transcribed verbatim, and translated into English where necessary. The transcripts were then transferred into and analysed using the NVivo qualitative analysis software version 12 (QSR International Pty Ltd). We used a thematic framework approach to identify, analyse and report patterns (themes) within data (Smith and Firth, 2011). To guide the analysis, we used an inductive approach to develop a codebook (Patton, 1990). The transcripts were then annotated by highlighting significant ideas. A thematic framework or codebook of themes was developed by co-authors, based on the key highlights and topics on the guides. All transcripts were re-read and coded for themes and sub-themes by two coders trained in qualitative data analysis. Analysis charts were then prepared for each thematic area and category of participants. We used these charts to identify common themes across participants and sites.

### Rigour and trustworthiness

We employed multiple strategies to enhance credibility, transferability, dependability and confirmability (Forero *et al.*, 2018; Johnson *et al.*, 2020). These included: (1) the use of multiple qualitative data sources with a variety of respondents (i.e. case narrative, IDIs and FGDs) to explore their views and experiences (credibility); (2) training and supervising field workers and ensuring that interviews were conducted in the local language respondents were comfortable with (dependability); and (3) translating transcripts to English by professional translators fluent in both languages (confirmability). In addition, four research team members individually read at least two transcripts from each site and type of interview to identify response categories for each question and created one codebook based on these themes. Any discrepancies in coding were discussed until consensus was reached. Finally, we followed the COREQ criteria in conducting the analysis and interpretation of the results (Tong *et al.*, 2007).

## Results

### Number of SYIs linked with PHCFs

Table 2 shows the number of SYIs linked with PHCFs by CHVs from August 2021 to July 2022. CHVs recorded 12 552 live births, among which 81% (10 187) were assessed for danger signs. Of those assessed, 1176(12%) were identified with PSBI danger signs and referred to the nearest PHCFs for care. Of those identified with PSBI danger signs, 820 (70%) accepted referral and sought care. The proportion that accepted referral was higher in Rongo sub-county (97%) compared to 62% and 61% for Bunyala and Teso North sub-counties, respectively. A majority of SYIs treated at PHCFs were followed up on day 2 (93%) and day 4 (77%). Eight deaths were recorded, all from Busia site (3 from Bunyala and 5 from Teso North).

**Table 2.** Number of SYIs linked with PHCFs by CHVs in Busia and Migori Counties (August 2021–July 2022)

	Rongo sub-county (Migori, *n* (%))	Bunyala sub-county (Busia, *n* (%))	Teso North sub-county (Busia, *n* (%))	Total, *n* (%)
Number of CHVs	395	189	143	395
Total number of newborns (0–2 months) within the period	3036	4194	5322	12 552
Number of newborns assessed (0–2 months) assessed by CHVs in the community	3036(100.0)	3140(74.9)	4011(75.4)	10 187(81.2)
Number of SYIs identified in the community and referred to the health facility by CHVs	271(8.9)	380(12.1)	525(13.1)	1176(11.5)
Number of SYIs referred by CHV and accepted referral (went to the health facility)	262(96.7)	236(62.1)	322(61.3)	820(69.7)
Number of SYIs with PSBI treated at PHCFs who received follow up assessment on day 2	246(93.9)	223(94.5)	291(90.4)	760(92.7)
Number SYI with PSBI treated at PHCFs who received follow up assessment on day 4	221(84.4)	186(78.8)	224(69.6)	631(77.0)
Number of SYIs that succumbed	0	3	5	8

Source: *COVID-19: Mitigating Neonatal Mortality Project Monitoring Data, 2022.*

**Figure 2.**
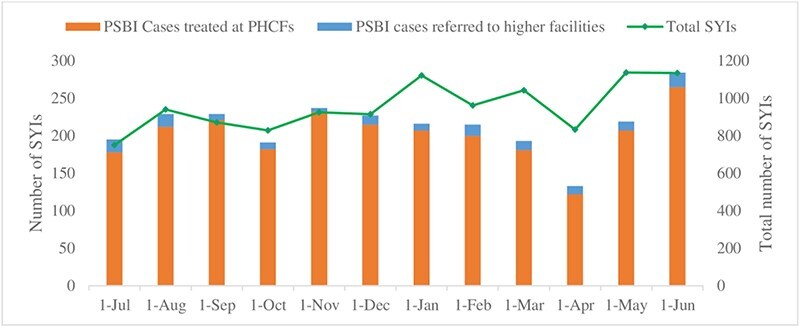
Number of sick young infants (0–59 days) care-seeking from PHCFs, including the total number of PSBI cases treated and referred, Busia and Migori Counties (July 2021–June 2022)

Service statistics showed a rising trend in the number of SYI treated at PHCFs over time, suggesting improved demand for SYI services in the community during the intervention period (Figure 2). The stacked columns are divided into PSBI cases treated at PHCFs or referred to higher level facilities by month. The chart shows an initial increase in the proportion of PSBI cases assessed and treated at the beginning of the intervention (July–October). However, there were fluctuations in some months, which may be attributable to the seasonality of birth events.

### Facilitators to community-facility linkage for case management of PSBI

#### CHVs’ relationship with community members and facilities

The relationship between CHVs and community members, as well as facilities, played a key role in strengthening the linkage between the community and facility for SYIs. Caregivers mentioned various positive aspects of CHVs that facilitated prompt linkage of SYI with facilities for the management of PSBI cases. For example, caregivers relied on CHVs in identifying danger signs such as fever, breastfeeding problems, difficulty breathing/cold, coughs and eye problems, which triggered timely care-seeking. In addition, the CHVs largely determined where care was sought for SYIs by referring cases identified in the community to nearby facilities. In most cases, proximity to facilities and cost of services determined where care was sought.

*When I realised the baby**’**s temperature was high, I called [Name of CHV]. She came and assessed the baby - checked the baby’s temperature and breathing pattern. She wrote a referral for me to take the baby to the hospital. [CN 02 Teso North]*

*Interviewer: What informed the decision on where to seek care? Respondent: It is the only nearest health facility. [name of facility] was very far. However, I delivered there and even after delivery, they told me to seek care at the nearest health facility, which is [name of facility] dispensary. [CN 05 Rongo]*

CHVs facilitated referral linkage for PSBI by accompanying the patient to the facility and conducting follow ups of treated SYI cases to monitor their progress towards full recovery. Priority was given to SYIs escorted to the facility by the CHVs. Thus, many caregivers preferred to go through CHVs to reduce waiting time for SYI services at the facility.

*They [caregivers of SYIs with PSBI] do not queue. …a CHV will just come and tell you that they have a patient and since we know the CHVs; they are our friends, you will tell them to bring the patient in for examination [IDI_Provider_01_Bunyala]*

Most caregivers, regardless of the site, expressed satisfaction with the SYI services and support provided by CHVs, such as identifying danger signs, referral to health facilities and information on where to seek care. Caregivers who were referred or escorted by CHVs received preferential treatment at the facility and did not have to wait in the queues to access SYI services.

*Yes, I was satisfied. I presented the referral form and got attention immediately at the waiting desk. Although I bought medicine at a pharmacy outside the facility, my child’s condition improved. I am happy that the CHV encouraged me to go to the hospital for my child’s treatment. With a referral card from CHV, even if the queue is long, you will be attended to ahead of other people. [CN 05 Bunyala]*

Similarly, facility-based providers noted the important role of CHVs in identifying, counselling and referring SYIs with PSBI. CHVs conducted home visits to educate caregivers about danger signs related to SYIs, thereby improving timely care-seeking and follow-up. Providers noted that CHVs needed to be supported, for example, with transport, to cover more households.

*Caregivers who fail to bring their babies for day 2 and day 4 reviews can be reached through CHVs in the community. So CHVs are doing a good job; they should be supported so that they can reach clients easily. [IDI_Provider_05_Rongo]*

### Availability of a CHV desk

The availability of a CHV desk (a link desk located at facilities, staffed by a CHV trained to coordinate and support clients and their families to ensure that their linkage to treatment is smooth) facilitated the community-facility linkage for managing PSBI cases in several ways. First, caregivers were assisted in navigating various points of care within facilities, thereby reducing the time spent in care-seeking. Second, the CHV desks maintained a roster with CHVs’ phone numbers, which facilitated the linkage of SYIs treated at the facility with CHVs in their catchment areas, ensuring effective follow-up in the community. Additionally, in some facilities, CHV stationed at the link desk performed other task-shifting activities such as taking weights and temperatures at triaging sections. This not only expedited the process but helped reduce the provider workload.

*The CHV desk helps the facility in that when the clients come in, they get one direction. Clients accompanied feel comfortable in the presence of CHVs who are familiar with them since they fear the hospital environment. CHV accompany and direct them. It also links the community and the facility because we involve them in what we do. They help us weigh the babies because sometimes the persons stationed there may not be around, and they give directions on where a patient is supposed to go next. [IDI_Provider_01_Rongo]*

### Use of mobile app technology

Use of a mobile app technology facilitated the community-facility linkage for PSBI cases across the study sites. For example, most CHVs noted that they could quickly link household registration information using the mobile app, unlike the paper-based manual system, where data loss was common. The mobile app also facilitated their observation of sick babies and assisted them in determining the necessary action to take, such as deciding whether the condition required the baby to be referred. Moreover, most CHVs mentioned that the mobile app helped them provide accurate danger signs and antibiotic dosage information to caregivers.

*With the [mobile] application, work has been easier. As you open the app, I select your name [household], which will indicate that [name] is 3 years or 2 months old. The app will guide and indicate that the baby is of a certain month. If the baby has surpassed 2 months, it will also inform you. So, it’s as if the phone is talking. It [the mobile app] also guides the dose, the time for administration, and the measurements. It has therefore made work easier tracking and following up on [PSBI] cases [FGD_CHV_01_Bunyala]*

*Comcare [the mobile app] will remind you to make a follow-up. It makes it easy for us to know the duties we have not performed for the baby in the community. Making a referral is also easy, we just click, and it asks us if there are any danger signs and takes us to the Referrals, then Tasks, then Follow up. It also tells if the baby’s condition has improved or not. [FGD_CHVs_02_Rongo]*

### Capacity strengthening

Training, which included workshops, on-job mentorship and continuous medical education, along with monthly supportive supervision, played a key role in facilitating community-facility linkage for the management of PSBI. Most CHVs and facility-based providers across the study sites acknowledged that the training they received at the beginning of the project implementation enhanced their capacity to educate caregivers about the danger signs, assess newborns for PSBI danger signs, refer them to the facility for treatment and conduct necessary follow ups.

*We learnt that if we visit a household, we should assess if a child has a cough, fast breathing, or chest in-drawing, which are possible signs of pneumonia. We were taught that if we visit a household and get a baby with chest in-drawing, we should refer them to a facility immediately. [FGD_CHVs_02_Rongo]*

Most providers and CHVs valued the supervision, mentoring and training sessions they received, which contributed to the fidelity in implementing PSBI guidelines. In addition, monthly review meetings were important for identifying early challenges and informing subsequent supervision and refresher training.

*In my opinion, the supervision was very helpful because it gave us morale. The community appreciates that we work on behalf of the government when the CHEW [community health extension worker] visits us. Our clients treat us with respect, and we also have some confidence. [FGD_CHV_02_Teso North]*

*Yes, it was helpful because after the talks, she [CHEW] told me the area where I did not do well and how it was supposed to be done. [FGD_CHVs_02_Rongo]*

### Availability of CHVs’ tools

The availability of essential tools to CHVs such as the MOH 100 referral form, digital thermometer, pulse oximeter, PSBI management checklist and danger signs job-aid facilitated the community-facility linkage for PSBI services as highlighted in the excepts below.

*We have the MoH 100 that we use for referrals. When I refer [SYI], I give two copies to the mother to take to the hospital and one copy that remains in the booklet. They then return with one copy that I go to check within 24 hours of return from the hospital, and I will proceed with that until the baby heals. [FGD_CHVs_02_Rongo]*

*When we carry it[job-aid] to the field, we can look up the danger signs and even show the mother or caregiver how they are. When she[caregiver] sees them, you know some signs they do not know, but she might know them when they see even on a picture. So, it also helps us when doing assessments and referrals. [FGD_CHV_01_Teso North]*

### Barriers to community-facility linkage for management of PSBI

Challenges related to the health system including inadequate providers, stockout of essential commodities and supplies and lack of transport/ambulance hindered community-facility linkage for PSBI management. Additionally, there were challenges associated with household-or individual factors, such as caregivers refusing to take referrals.

### Inadequate providers

Facilities that had few providers experienced challenges related to heavy workload, which affected the linkage for PSBI services. This challenge was evident during the referral process, both to higher level facilities and when referring back to the community. The heavy workload led some providers to choose not to document PSBI cases or complete copies of the MOH-100 form, which served as feedback for the referring CHVs. In such instances, CHV relied on verbal feedback from caregivers to update the details in the mobile app.

*…so you can imagine you are loaded with so many patients, and a child presents with convulsions, and you are not able to document everything like pre-referral treatment, you just stabilise the child, and when the convulsions cease, you call the referral facility and talk over the phone- I have given this and that [intervention]; because you do not have that time to document. [IDI_Provider_01_Teso North]*

Shortage of providers in some facilities led to long waiting times at facilities, causing delays in receiving services for SYIs and affected the acceptability and adherence to PSBI guidelines.

*Providers should give prompt service to caregivers with SYI instead of keeping them on the queue for an extended time. … I think they should consider serving mothers faster to motivate them and encourage them to continue seeking care for babies when they need it. [Caregiver 05, Rongo]*

### Stockout of essential drugs

The persistent challenge of stockout of essential drugs for managing PSBI affected the community-facility linkage for the management of PSBI when a referral was not feasible.

*So, you can prescribe for the patient, and when she goes to the chemist, she is given something different than what you have written. So, yeah, and the facility has not been supplied with or able to get any commodities like these antibiotics for quite some time. [IDI_Provider_01_Bunyala]*

*Sometimes, there is no Amoxicillin DT in stock. It is a big challenge to deal with. I could tell a parent to buy, but she just disappears if she does not have money. Sometimes, caregivers go to a specific facility and are told to buy medicine. Then, after two days, they return when the infection is worse. So, that is the challenge when we miss them because we give them for free. [IDI_Provider_05_Rongo]*

### Lack of transport/ambulance for referral

Lack of transport for referral posed a challenge for referrals and had an impact on the community-facility linkage for PSBI treatment. Insufficient transport was compounded by factors such as long distance to the referral facilities, poor road infrastructure and the absence of ambulatory services.

*The problem comes when you want to transfer a mother to another facility. Unless you have a vehicle and go with the mother in it or take a boda boda to leave the mother at the facility, they will disappear and not go for the referral. [IDI_Provider_02_Rongo]*

### Caregiver failure to accept the referral

Some caregivers declined referrals due to various factors, including lack of money to cater for transport and treatment expenses.

*Regarding referral, it is a challenge because of socioeconomic status. Many people here are poor, so they cannot afford to board a vehicle to another facility. They are also unable to buy medication. So maybe a patient is using a particular medication, and you would prefer that she changes to another; that becomes a challenge. It becomes a challenge because they cannot buy it [Amoxil] even after prescribing it. [IDI_Provider_01_Bunyala]*

## Discussion

As countries scale up the implementation of WHO’s PSBI guidelines, there is much to learn about creating effective and sustainable linkages between communities and health facilities. This is important for institutionalizing the management of PSBIs at PHCFs when referral is not feasible. This study explored facilitators and barriers to community-facilities linkages for implementing WHO’s guideline for case management of PSBIs in SYIs (0–59 days old) when a referral is not feasible or acceptable in Kenya.

Our study demonstrated that trained CHVs, when supervised and incentivized to work within the existing health system, can effectively coordinate within their communities to assess SYIs with PSBI, and initiate appropriate linkage with PHCFs for timely treatment. Although the coverage estimates from the monitoring data may not precisely reflect the actual population-level estimate, CHVs assessed almost all newborns in their respective CHUs: 1176 (12%) of newborns were identified with PSBI danger signs, a range consistent with documented PSBI incidence rates in other studies (Seale *et al.*, 2014). As shown in the literature, CHVs are instrumental in bridging community and clinical services for high-risk conditions (Levitt *et al.*, 2015). In our case, they played a critical role in case management of PSBI in young infants (0–59 days old) by assessing and referred SYIs to PHCFs. However, the proportion accepting referrals may be influenced by contextual and programmatic factors. For instance, Bunyala and Teso North sub-counties in Busia County, characterized by vast rural areas and poor road infrastructures, showed lower referral acceptance compared to Rongo sub-county in Migori County. In addition, Rongo had a high CHV-to-household ratio with fewer households to visit. Referral of SYIs to facilities by CHVs enables many families to access prompt, life-saving care, ultimately improving newborn health outcomes (Levitt *et al.*, 2015; Huda *et al.*, 2023).

Several factors facilitated successful linkage for PSBI treatment. The relationship between CHVs and community members as well as facilities was key to community-facility linkage for PSBI care. Existing research has emphasised the significance of a strong relationship between CHVs, communities and health facilities in ensuring compliance with referral and uptake of maternal and newborn health services (Nsibande *et al.*, 2013). CHVs are respected and trusted by the community members, given their status as members of the community they serve. Furthermore, CHVs are more easily accessible to community members compared to health facilities, serving as the primary point of contact for health information and linkage with clinical care (Khanal *et al.*, 2011; Lohr *et al.*, 2018; Avan *et al.*, 2021). The majority of caregivers of SYIs interviewed reported consulting with the CHVs first, who then facilitated the linkage with health facilities. The community-facility linkage was further strengthened through the CHV desk at some of the study facilities. These desks coordinated case management, tracked SYIs and served as a bridge between the community and facility. Upon receiving SYIs from the community, the CHV desk registered SYIs for clinical evaluation, facilitating their linkage to other health services and enabling home-based follow-up.

Several implementation strategies were also identified as facilitators. For instance, training and supportive supervision proved to enhance community-facility-linkage for PSBI care. When equipped with proper training and support, CHVs can be potential catalysts for improved newborn care and improve child health outcomes through early identification and timely referral to health facilities (Kayemba Nalwadda *et al.*, 2013). Similar to lessons learned from an earlier PSBI implementation research in a different setting in Kenya, our study revealed that building the capacity of CHVs is critical in creating awareness of PSBI danger signs and facilitating prompt identification and referral of SYIs to health facilities(Liambila *et al.*, 2023).

Leveraging CHVs and emerging mobile technologies can potentially help improve identification rates of SYIs in the community and ensure appropriate referral in low-resource settings. The PSBI II intervention achieved a modest referral acceptance rate (70%) to PHCFs for newborns with PSBI danger signs. Similar to other studies in SSA (Källander *et al.*, 2013; Gibson *et al.*, 2017), the mobile app technologies applied by the implementing partners improved the identification, referral and tracking of PSBI cases by CHVs and facilitated linkage with PHCFs. Thus, combining CHVs’ efforts with mobile digital strategy enhanced the fidelity of implementing WHO’s PSBI guidelines by ensuring reliable real-time data and timely interventions. Through the mobile apps, CHVs accurately assessed SYIs and referred PSBI cases promptly. In addition, the mobile app provided CHVs with reminders for household visits and follow-ups of PSBI cases to assess treatment compliance and the recovery process, thereby improving the continuum of PSBI care.

Our finding confirms documented barriers and challenges to community-facility linkage for newborn health services, many of which were beyond the scope of the PSBI II project. These challenges include health system-related barriers such as inadequate providers and stockout of essential commodities (Nair *et al.*, 2014; Sundararajan *et al.*, 2015; Miller *et al.*, 2021). Inadequate providers led to delays in receiving care due to congestion and long queues at PHCFs. Additionally, stockout of essential drugs often led to dissatisfaction with SYI services among caregivers. Other barriers to community facility linkage were individual or household related. Long distances and financial costs limit linkage for PSBI care by creating barriers to the accessibility and affordability of referral services at the health facility. These challenges may have been exacerbated by the impact of the COVID-19 pandemic. In particular, the COVID-19 pandemic and associated control measures negatively impacted access to child health services due to disruptions in the health workforce, supply chains and decreased care-seeking due to fear of infection risk at health facilities and financial inability to pay for healthcare services (Kimani *et al.*, 2020; Oluoch-Aridi *et al.*, 2020)

### Strength and limitations

The main strength of our study is that we captured caregivers’ personal experiences with linkage to PSBI care. However, some limitations may influence our findings. First, study participants were purposively identified based on their role in the intervention, which potentially may lead to positive views. However, some participants reported challenges, therefore, the selection process did not appear to bias the qualitative data collection towards only those with positive views. Second, this study was implemented in two counties of Western Kenya, where a robust CHV programme had been implemented. Therefore, the findings might not be generalizable to other geographic regions of the country. While the study that generated data for this paper was focused on mitigating the impact of COVID-19 on neonatal mortality, the pandemic and its preventive measures may have disrupted service delivery and negatively affected the performance of primary care systems to deliver quality newborn health services in the study sites. Nonetheless, our study findings will help in understanding the barriers and facilitators to expansion of PSBI treatment in low-resource settings and generate learnings to optimize PSBI guideline implementation during COVID-19 and similar crises.

## Conclusion

Leveraging well trained, supervised and incentivized CHVs, along with emerging mobile technologies, and establishing CHV desks at primary health facilities strengthen linkages between communities and facilities. This, in turn, improves access to PSBI care at PHCFs and community follow-up.

## Data Availability

The data underlying this article cannot be shared publicly due to the privacy of individuals that participated in the qualitative interviews. Request to access the data may be sent to Population Council, Dataverse, email: publications@popcouncil.org, for information on data access.

## Reflexivity statement

The authors include five females and two males and span multiple levels of seniority. While two of the authors have expertise in clinical medicine and specialize in child health in Kenya and the global south, the first four authors have experience conducting implementation research studies in Kenya. The first and second authors have extensive experience conducting qualitative fieldwork in Kenya, especially in Coast and Western regions.
